# Feline malignant lymphoma in an uncommon location as a differential diagnosis for neurological disease

**DOI:** 10.1177/20551169241300815

**Published:** 2025-02-15

**Authors:** Maximilien Lépine, Sarah Schmitz, Svenja Körber, Kernt Köhler

**Affiliations:** 1 Institute of Veterinary Pathology, Justus Liebig University, Giessen, Germany; 2 Clinic for Small Animals, Surgical Department, Department of Veterinary Clinical Sciences, Justus Liebig University, Giessen, Germany

**Keywords:** Lymphoma, glomus caroticum, paraganlioma, chemodectoma

## Abstract

**Case summary:**

A 12-year-old male castrated domestic shorthair cat exhibited right Horner’s syndrome, right facial nerve paresis, difficulty swallowing, coughing, gait abnormalities and weight loss. Despite prior unspecific treatment by a primary care veterinarian with cortisone and antibiotics, the cat’s condition worsened, culminating in tetraparesis and right hemispasms. Imaging studies, including CT and MRI, identified a mass extending from the carotid body into the neurocranium, causing displacement of adjacent brain structures and meningeal contrast uptake. Histopathology confirmed a malignant B-cell lymphoma. Differential diagnoses are explored, with a particular focus on carotid body tumours, which originate from the chief cells of the carotid body. These neoplasias are rare in non-human primates, dogs, cats and horses, possibly influenced by genetic predisposition and environmental factors such as hypoxia.

**Relevance and novel information:**

Carotid body tumours are rare in cats, as they are in other animal species. Although lymphomas are the most common feline neoplasms, to our knowledge, no previous case of a B-cell lymphoma in the carotid body has been described in the feline species to date. This case underscores the importance of considering rare and common neoplastic entities in feline patients with atypical clinical presentations and locations. Thereby highlighting the diagnostic challenges in veterinary oncology.

## Introduction

Lymphoma is a common neoplasm and the most common haemopoietic neoplasm in cats.^1^ Lymphomas mainly arise from lymph nodes but can have their origins in a variety of other organs.^2^ Their anatomical location is one basis for classification. The main locations of lymphoma include the multicentric, thymic/mediastinal, gastrointestinal, cutaneous, extranodal and central nervous system (CNS). This classification does not include lymphomas in uncommon locations, such as the carotid body.^3^ Of all lymphomas, approximately 13% are located in the CNS, mostly as part of a multisystemic neoplastic disease.^4^

In addition, lymphoid neoplasms are classified based on their ultrastructural morphology and their cellular origin, which is confirmed through immunohistochemical staining^5^ or through antigen aberrancies in flow cytometry.^6,7^ These tools are essential for the explicit diagnosis of lymphomas, as a large number of subtypes play a role in this group, which are morphologically uniform but phenotypically different. Lymphomas can be differentiated between B-cell and T-cell, and there are other uncommon aberrant phenotypes.^2^ In dogs, 60–70% of lymphomas are of B-cell origin; however, in cats, enteric T-cell lymphomas make up approximately 50% of all feline lymphomas since the vaccination for feline leukaemia virus (FeLV) is broadly available.^8^

The most established markers are CD3 for the detection of T cells and CD79a for the detection of B cells. CD20 and Pax-5^9^ can be used as further markers for the detection of B cells in cats. However, markers based on B220, a B-cell specific member of the T200 glycoprotein family, have also been established as B-cell specific markers.^2^ These are used in CD45R antibodies.^10^ Other antibodies that can be used to further classify lymphomas, for example, are CD11b, CD11c, CD14, CD21 and CD34.^2^

The glomus caroticum, also known as the carotid body or paraganglion intercaroticum, is located at the bifurcation of the common carotid artery. It is approximately 1.5 mm long, 1 mm wide and 0.5 mm thick. On cross section, it is spindle-shaped or triangular.^11^ Neoplastic diseases of the carotid body are rare, but the aortic and carotid bodies are the most common sites of paragangliomas in dogs^12,13^ and cats.^14^ However, they have been described in the glomus pulmonale,^15^ in the adrenal gland^16^ and in the jugulotympanic paraganglia^17^ of dogs. In cats, chemodectomas are reported in the carotid body,^18^ in the aortic body,^14,19–26^ in the vena cava,^27^ in the cauda equina^28^ and, recently, in the glomus pulmonale^24^ and in the orbita.^29^ Metastases occur in approximately 3.3% of carotid body tumours^3^ and have been found in the lung,^28^ myocardium, pericardium, cranial mediastinal lymph nodes,^19^ diaphragm and intercostal muscles of cats.^30^

Immunostaining has been established for the detection of paragangliomas in humans. One study showed that S-100 protein is present in all carotid body tumours. In addition, most tumours also exhibit neuron-specific enolase (NSE), chromogranin A (CHA), serotonin (SER) and synaptophysin (SYN).^31,32^ In a feline aortic body tumour, the cells were immunohistochemically positive for CHA, for SYN and, faintly, for NSE, and negative for vimentin, cytokeratin, a smooth muscle actin, glial fibrillary acidic protein, thyroglobulin and calcitonin.^20^

## Case description

A 12-year-old male castrated domestic shorthair cat was admitted to the referral clinic with tetraparesis and right hemifacial spasms. The owner stated a 1-week history of right Horner’s syndrome, right facial nerve paresis, constant swallowing, coughing and dysphonia. Gait was abnormal with stumbling on the right hindlimb and the cat was not able to open its jaw to the maximum, noticed by the inability to take in larger pieces of food and the absence of yawning. Pretreatment administered 1 week before presentation in the referral clinic by a primary care veterinarian consisted of cortisone and an unknown systemic antibiotic. Owing to a lack of improvement, the cat was admitted to the clinic 2 weeks after the first evident clinical signs.

On physical examination, the cat presented as apathetic and in lateral recumbency. The cat showed generalised muscle wastage and a reduced body condition score of 2/9. The cat presented with an inability to open its mouth completely. During palpation of the throat, the cat swallowed multiple times and showed discomfort.

On neurological examination, the cat showed an inability to stand and walk, while motor function was preserved. Postural and positional reactions as well as proprioception was reduced on all limbs. Spinal reflexes were moderately excessive on the left forelimb and hindlimb. During evaluation of the cranial nerves, the cat showed normal visual functions, anisocoria with a miosis on the right, right enophthalmos, resulting in prolapse of the nictitating membrane, consistent with the preliminary report of a right Horner’s syndrome. Neuroanatomical location was suspected to be the cervical cord (C1-5) and/or the brainstem. Haematology and a biochemical analysis were unremarkable, as were paired radiographs of the thorax.

**Figure 1:**
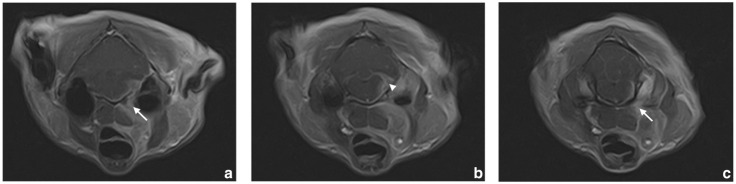
Set of MRI scans of the head. Starting from the region of the right medial retropharyngeal lymph node and carotid angle ([a,c] arrows), a space-occupying mass is visible, which extends towards the tympano-occipital fissure ([b] arrowhead)

MRI was performed and showed a space-occupying mass reaching from the carotid angle to the tympano-occipital fissure (Figure 1). The fissure was mildly dilated and the bone of the skull base showed low-grade sclerosis. The mass entered the neurocranium via the fissure. Within the neurocranium, a poorly circumscribed large mass with a diameter of approximately 4 mm was seen around the cerebellopontine angle, displacing the adjacent portions of the brainstem and cerebellum. There was a high degree of contrast enhancement in the intra- and extracranial regions of the mass and along the meningeal surface of the brainstem extending rostrally to the hypophyseal fossa. The meningeal contrast uptake was visible mainly on the right side, where it extended to the level of the flocculus. There was a high degree of contrast enhancement within the internal acoustic meatus along the facial nerve and vestibulocochlear nerve.

MRI findings suggested a neoplasia in the carotid body extending to the neurocranium. Owing to the poor prognosis and at the owner’s request, the cat was humanely euthanased.

**Figure 2:**
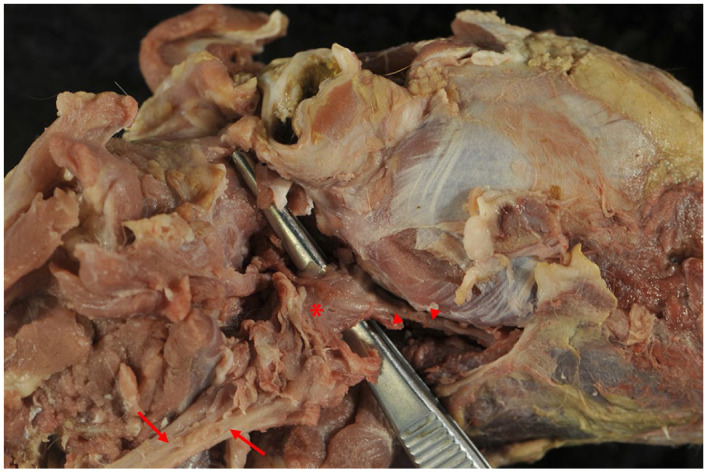
Right lateral view of the cranial neck and head. The mass (asterisk) is close to the common carotid artery (arrows) as well as the external carotid artery (arrowheads)

A full post-mortem examination was performed and in the region of the carotid angle, a 2 × 1.5 × 0.5 mm large firm mass with a homogenously pale pink and round cross section was detected (Figure 2). The bulla and its surrounding tissue did not reveal any macroscopic changes. Other organs and lymphoid tissue were unaffected.

**Figure 3:**
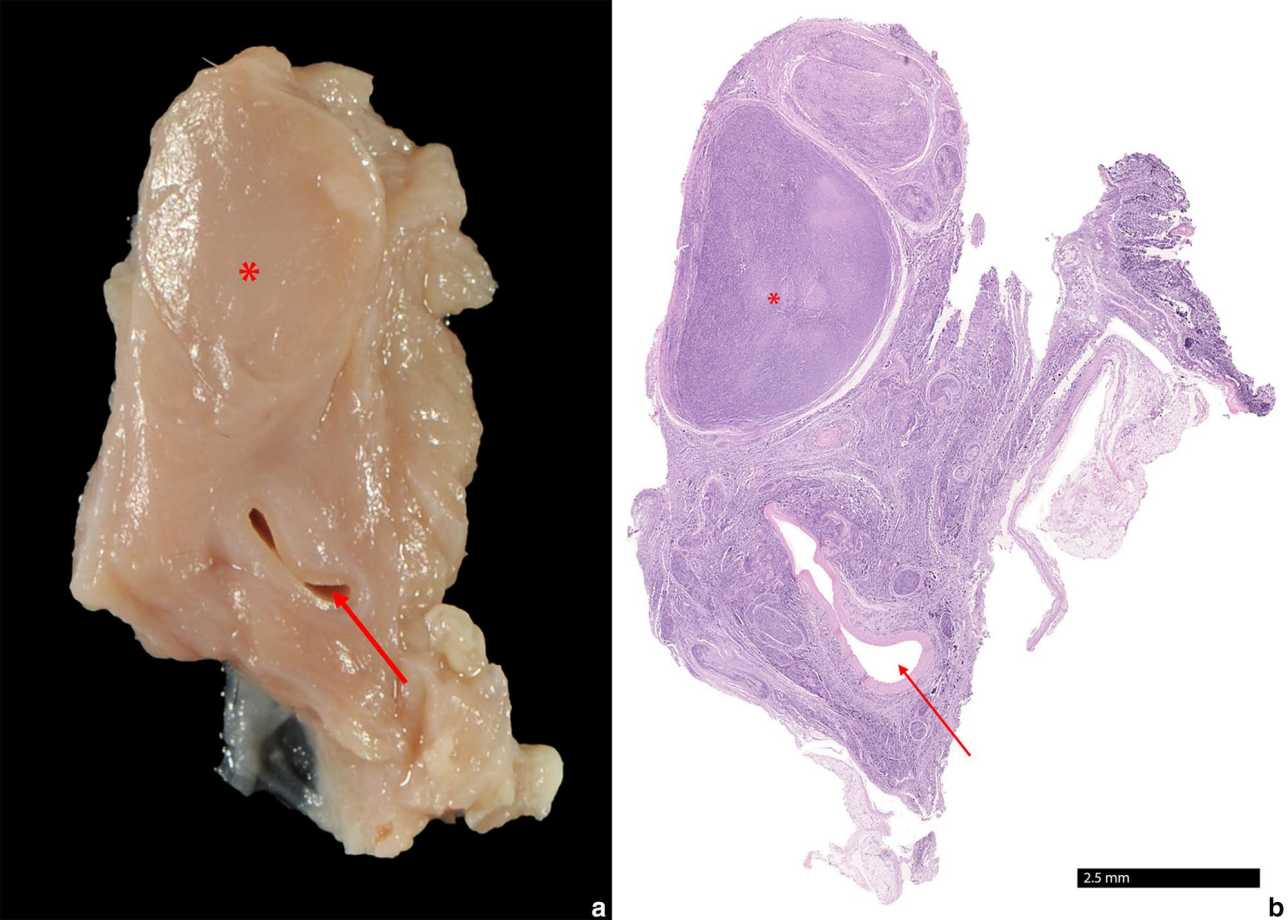
(a) A 2 × 1.5 × 0.5 mm large firm mass (asterisk) with a homogeneously pale pink and round cross section is located close to the common carotid artery (arrow). (b) Histologically, the mass is encapsulated and of high cellularity

**Figure 4:**
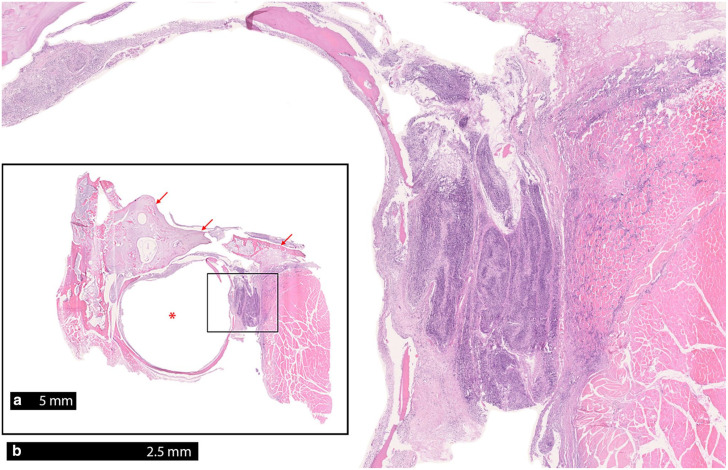
(a) There is evident cellular infiltration medially to the bulla (asterisk), but not in the ventral cranium (arrows). (b) Pronounced infiltration of the nervous tissue and parts of the skeletal muscles with monomorphic round cells (inset of panel [a])

**Figure 5:**
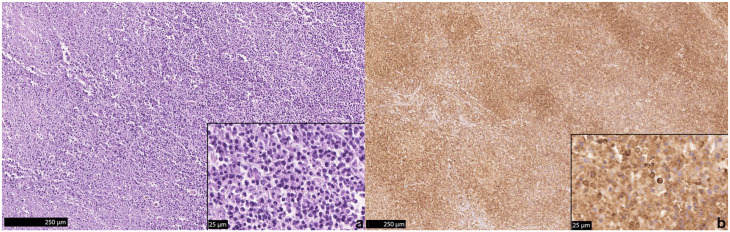
(a) The carotid body is infiltrated with a high number of monomorphic round cells. (b) The majority of cells is immunopositive for CD45R

Histologically, an infiltration with a high number of monomorphic round cells in the carotid body with additional pronounced infiltration of nervous tissue was observed (Figure 3). Adjacent to the bulla, multiple regions with pronounced infiltration of monomorphic round cells of nervous tissue, presumably the glossopharyngeal nerve, and portions of skeletal muscle were evident (Figure 4). Immunohistochemistry was performed using the primary antibodies listed in Table 1. Paraffin sections were deparaffinised in xylene and rehydrated through graded alcohols. Endogenous peroxidases were inhibited by incubation (30 mins) in methanol–hydrogen peroxide. Slides were labelled with corresponding antibodies. Chromogenic methods were either ABC (Avidin-biotin complex; Linaris Biologische Produkte) for CD45R, NSE, SYN, cytokeratin, vimentin or PAP (peroxidase-antiperoxidase; Dako/Agilent Technologies) for CD3, CHA, S-100 and thyroglobulin. For each antibody, a positive control was included. For negative controls, primary antibodies were replaced by non-reacting antibodies. The neoplastic cells were immunopositive for CD45R and a low number of cells were immunopositive for CD3 (Figure 5). CD45R has been shown to be a specific B-cell marker in cats, as it detects the epitope of the B220 antigen of the CD45 glycoproteins,^10,33–36^ resulting in the diagnosis of a malignant B-cell lymphoma.

**Table 1:** List of the antibodies used and results of immunohistochemical staining

Antibody	Origin, clone/polyclonal	Supplier	Dilution	Reactivity
CD3	Rabbit, polyclonal	Agilent Dako	1:100	(+) some interspersed cells
CD45R	Rat, clone B220 (Ly 5)	Cedarlane	1:1000	+
SMA	Mouse, clone 1A4	Agilent Dako	1:500	−
S-100 protein	Rabbit, polyclonal	Agilent Dako	1:400	−
NSE	Mouse, clone BBS/NC/VI-H14	Agilent Dako	1:100	−
Cytokeratin	Mouse, clone AE1/AE3	Agilent Dako	–	−
SYN	Mouse, clone SY 38	Agilent Dako	1:100	−
CHA	Rabbit, polyclonal	Origene	1:10	−
Vimentin	Mouse, clone V9	Agilent Dako	1:50	−
Thyroglobulin	Rabbit, polyclonal	Agilent Dako	–	−
Glial fibrillary acidic protein (GFAP)	Rabbit, polyclonal	Agilent Dako	1:500	(+) some interspersed cells

CHA = chromogranin A; GFAP = glial fibrillary acidic protein; NSE = neurone-specific enolase; SMA = smooth muscle actin; SYN = synaptophysin

## Discussion

The clinical signs, the unique location and the regional spread of the tumour in the MRI led clinicians to the first differential diagnosis of a paraganglioma of the glomus caroticum. In human medicine, invasion of nearby nerves or tissue adjacent to the base of the skull, as seen in this case, has been reported.^37^ Even though the neoplasm in this case turned out to be a malignant B-cell lymphoma, several differentials to neoplasms in the region near the angle of the mandible should be considered. As a result of the anatomic proximity, tumours of the mandibular lymph nodes, the medial retropharyngeal lymph nodes, the mandibular gland and the parotid gland would be valid differential diagnoses for tumours in this region, which are more frequent than tumours of the carotid body. However, these locations could be excluded as the origin of the tumour, owing to the performed diagnostic imaging.

During the post-mortem examination, the mass could be confirmed to be located in the glomus caroticum and the surrounding tissues, such as glossopharyngeal nerve and skeletal muscle. Through histopathology and immunohistochemical staining, the neoplasm was diagnosed as diffuse large B-cell lymphoma (DLBCL). According to the Revised European-American Lymphoma (REAL) classification of lymphoid neoplasms adopted by the World Health Organization, the DLBCL is part of the mature (peripheral) B-cell neoplasm. DLBLC is the most common lymphoma in most domestic species.^38^ In cats, B-cell lymphoma is common and many of them were located in the mediastinum,^39^ but were also found in the upper respiratory tract, segments of the bowel or were multricentric.^1,39–41^ Therefore, this case represents an outlier of a B-cell lymphoma in an unusual location.

In the present case, a tumour arising from the carotid body is most probable. On the other hand, a primary CNS lymphoma extending towards the carotid body is a possibility, but the prevalence of primary CNS lymphomas is very low, at only 3% of primary CNS tumours,^42^ with even less showing extension into the extracranial tissues. Extension into extracranial tissues has been reported in the nose in feline patients,^43^ but none can be found along the tympano-occipital fissure; however, there are reports of tumours originating from the carotid body invading nearby tissues in humans.^37^ In addition, the intracranial changes described in diagnostic imaging could not be histologically confirmed, reinforcing the assumption of the glomus caroticum being the origin of the tumour.

Other neoplasms of the nervous system have been ruled out and the unique dispersion of the lymphoma from the carotid body along the glossopharyngeal nerve with probable invasion of the neuropil can be held accountable for the observed signs.

## Conclusions

The present case report describes the clinical findings, diagnostic imaging and pathohistological changes in a cat with a tumour of the glomus caroticum. Even though the neoplasm turned out to be a malignant lymphoma, the unique location, the infiltration along nervous tissue and the absence of neoplastic cells in other examined organs, led at first to the assumption of a paraganglioma in the glomus caroticum. Therefore, this case shows the importance of keeping a common neoplasm at the top of the differential diagnoses.

## References

[1] Moore PF Rodriguez-Bertos A Kass PH. Feline gastrointestinal lymphoma: mucosal architecture, immunophenotype, and molecular clonality. Vet Pathol 2012; 49: 658–668. 21505197 10.1177/0300985811404712

[2] Meuten DJ (ed). Tumors in domestic animals. 5th ed. Ames, IA: John Wiley & Sons, 2017.

[3] Maxie MG. Jubb, Kennedy, and Palmer’s pathology of domestic animals: Volume 3. 6th ed. St Louis, MO: Elsevier, 2016.

[4] Mandara MT Motta L Calò P. Distribution of feline lymphoma in the central and peripheral nervous systems. Vet J 2016; 216: 109–116. 27687936 10.1016/j.tvjl.2016.07.013

[5] Valli VE (ed). Histological classification of hematopoietic tumors of domestic animals. Vol 8. Armed Forces Institute of Pathology, 2002.

[6] Martini V Poggi A Riondato F , et al. Flow-cytometric detection of phenotypic aberrancies in canine small clear cell lymphoma. Vet Comp Oncol 2015; 13: 281–287. 23721515 10.1111/vco.12043

[7] Martini V Bernardi S Marelli P , et al. Flow cytometry for feline lymphoma: a retrospective study regarding pre-analytical factors possibly affecting the quality of samples. J Feline Med Surg 2018; 20: 494–501. 28675320 10.1177/1098612X17717175 PMC11104065

[8] Vezzali E Parodi AL Marcato PS , et al. Histopathologic classification of 171 cases of canine and feline non-Hodgkin lymphoma according to the WHO. Vet Comp Oncol 2010; 8: 38–49. 20230580 10.1111/j.1476-5829.2009.00201.x

[9] Raskin RE Vickers J Ward JG , et al. Optimized immunocytochemistry using leukocyte and tissue markers on Romanowsky-stained slides from dogs and cats. Vet Clin Pathol 2019; 48: 88–97. 31347181 10.1111/vcp.12759

[10] Monteith CE Chelack BJ Davis WC , et al. Identification of monoclonal antibodies for immunohistochemical staining of feline B lymphocytes in frozen and formalin-fixed paraffin-embedded tissues. Can J Vet Res 1996; 60: 193–198. 8809382 PMC1263832

[11] Nickel R Schummer A Seiferle E. Nervensystem, Sinnesorgane, endokrine Drüsen. 4th ed. Stuttgart: Parey Verlag, 2003.

[12] Johnson KH. Aortic body tumors in the dog. J Am Vet Med Assoc 1968; 152: 154–160.

[13] Hayes HM Sass B. Chemoreceptor neoplasia: a study of the epidemiological features of 357 canine cases. Zentralbl Veterinarmed A 1988; 35: 401–408. 2844038 10.1111/j.1439-0442.1988.tb00052.x

[14] Tillson DM Fingland RB Andrews GA. Chemodectoma in a cat. J Am Anim Hosp Assoc 1994; 30: 586–590.

[15] Herrero BA Ecklund AE. Primary tumor of the glomus pulmonale producing pulmonary stenosis in a Boston terrier. Am Heart J 1967; 73: 188–194. 4289368 10.1016/0002-8703(67)90147-0

[16] Dorn A Theuring F Dittert R , et al. A polypeptide immunoreactive nonchromaffin paraganglioma in the periglandular connective tissue of glandula suprarenalis of a dog. A case report. Exp Pathol 1985; 27: 99–104. 2987019 10.1016/s0232-1513(85)80046-3

[17] Cooley AJ Fox LE Duncan ID , et al. Malignant jugulotympanic paraganglioma in a dog. J Comp Pathol 1990; 102: 375–383. 2365852 10.1016/s0021-9975(08)80159-4

[18] Yates WD Lester SJ Mills JH. Chemoreceptor tumors diagnosed at the Western College of Veterinary Medicine 1967–1979. Can Vet J 1980; 21: 124–129. 6249479 PMC1789750

[19] Buergelt CD Das KM. Aortic body tumor in a cat. A case report. Pathol Vet 1968; 5: 84–90. 5690463 10.1177/030098586800500111

[bibr20-20551169241300815] Paltrinieri S Riccaboni P Rondena M , et al. Pathologic and immunohistochemical findings in a feline aortic body tumor. Vet Pathol 2004; 41: 195–198. 15017037 10.1354/vp.41-2-195

[bibr21-20551169241300815] Del Busto I Stiborova K Villiers E , et al. Aortic chemodectoma causing a lymphocyte-rich effusion in a cat. Vet Rec Case Rep 2018; 6. DOI: 10.1136/vetreccr-2018-000620.

[bibr22-20551169241300815] Fossum TW Miller MW Rogers KS , et al. Chylothorax associated with right-sided heart failure in five cats. J Am Vet Med Assoc 1994; 204: 84–89. 8125826

[bibr23-20551169241300815] Caruso KJ Cowell RL Upton ML , et al. Intrathoracic mass in a cat. Vet Clin Pathol 2002; 31: 193–195. 12447782 10.1111/j.1939-165X.2002.tb00302.x PMC7164224

[bibr24-20551169241300815] Saunders R Kraipowich N Marshall HC. Intracardiac malignant nonchromaffin paraganglioma (chemodectoma) in a cat. J Vet Cardiol 2021; 37: 1–7. 34399378 10.1016/j.jvc.2021.07.002

[bibr25-20551169241300815] Willis R Williams AE Schwarz T , et al. Aortic body chemodectoma causing pulmonary oedema in a cat. J Small Anim Pract 2001; 42: 20–23. 11219818 10.1111/j.1748-5827.2001.tb01978.x

[26] Hansen SC Smith AN Kuo KW , et al. Metastatic neuroendocrine carcinoma of aortic body origin in a cat. Vet Clin Pathol 2016; 45: 490–494. 27564688 10.1111/vcp.12392

[27] Martinez I Brockman D Purzycka K. Caval chemodectoma in a cat. JFMS Open Rep 2022; 8. DOI: 10.1177/20551169221106990. 10.1177/20551169221106990 PMC9260574 35811937

[28] Davis WP Watson GL Koehler LK , et al. Malignant cauda equina paraganglioma in a cat. Vet Pathol 1997; 34: 243–246. 9163884 10.1177/030098589703400313

[29] Leonardi L Rizac RI Pettinari I , et al. A first case report of orbital extra-adrenal paraganglioma in cat. Vet Sci 2021; 8: 86. DOI: 10.3390/vetsci8050086. 34068893 10.3390/vetsci8050086 PMC8156548

[30] George C Steinberg H. An aortic body carcinoma with multifocal thoracic metastases in a cat. J Comp Pathol 1989; 101: 467–469. 2558129 10.1016/0021-9975(89)90030-3

[31] Johnson TL Zarbo RJ Lloyd RV , et al. Paragangliomas of the head and neck: immunohistochemical neuroendocrine and intermediate filament typing. Mod Pathol 1988; 1: 216–223. 2467285

[32] Kimura N Sasano N Yamada R , et al. Immunohistochemical study of chromogranin in 100 cases of pheochromocytoma, carotid body tumour, medullary thyroid carcinoma and carcinoid tumour. Virchows Arch A Pathol Anat Histopathol 1988; 413: 33–38. 3131955 10.1007/BF00844279

[33] Flatland B Fry MM Newman SJ , et al. Large anaplastic spinal B-cell lymphoma in a cat. Vet Clin Pathol 2008; 37: 389–396. 19055573 10.1111/j.1939-165X.2008.00049.x

[bibr34-20551169241300815] Henrich M Bauknecht A Hecht W , et al. Lack of Bcl-2 expression in feline follicular lymphomas. J Vet Diagn Invest 2019; 31: 809–817. 31585524 10.1177/1040638719877916 PMC6900727

[bibr35-20551169241300815] Kipar A May H Menger S , et al. Morphologic features and development of granulomatous vasculitis in feline infectious peritonitis. Vet Pathol 2005; 42: 321–330. 15872378 10.1354/vp.42-3-321

[36] Obert LA Hoover EA. Relationship of lymphoid lesions to disease course in mucosal feline immunodeficiency virus type C infection. Vet Pathol 2000; 37: 386–401. 11055861 10.1354/vp.37-5-386

[37] Lack EE. Atlas of tumor pathology: tumors of the adrenal gland and extra-adrenal paraganglia. Washington, DC: Armed Forces Institute of Pathology, 1997.

[38] Valli VE. Veterinary comparative hematopathology. Ames, IA: Blackwell Pub, 2007.

[39] Sato H Fujino Y Chino J , et al. Prognostic analyses on anatomical and morphological classification of feline lymphoma. J Vet Med Sci 2014; 76: 807–811. 24521793 10.1292/jvms.13-0260 PMC4108762

[bibr40-20551169241300815] Chino J Fujino Y Kobayashi T , et al. Cytomorphological and immunological classification of feline lymphomas: clinicopathological features of 76 cases. J Vet Med Sci 2013; 75: 701–707. 23337319 10.1292/jvms.12-0246

[41] Valli VE Jacobs RM Norris A , et al. The histologic classification of 602 cases of feline lymphoproliferative disease using the National Cancer Institute working formulation. J Vet Diagn Invest 2000; 12: 295–306. 10907857 10.1177/104063870001200401

[42] Vandevelde M. Veterinary neuropathology: essentials of theory and practice. Oxford: Wiley-Blackwell, 2012.

[43] Palus V Volk HA Lamb CR , et al. MRI features of CNS lymphoma in dogs and cats. Vet Radiol Ultrasound 2012; 53: 44–49. 22093168 10.1111/j.1740-8261.2011.01872.x

